# Quantification of Plasma 8-Isoprostane by High-Performance Liquid Chromatography with Tandem Mass Spectrometry in a Case-Control Study of Lung Cancer

**DOI:** 10.3390/ijerph191912488

**Published:** 2022-09-30

**Authors:** Lin Ma, Dongxiao Sun, Guangli Xiu, Philip Lazarus, Anil Vachani, Trevor M. Penning, Alexander S. Whitehead, Joshua E. Muscat

**Affiliations:** 1 State Environmental Protection Key Lab of Environmental Risk Assessment and Control on Chemical Processes, School of Resources & Environmental Engineering, East China University of Science and Technology, Shanghai 200237, China; 2 Department of Public Health Sciences, Pennsylvania State College of Medicine, Hershey, PA 17033, USA; 3 Department of Pharmacology, Mass Spectrometry Core Facility, The Pennsylvania State College of Medicine, Hershey, PA 17033, USA; 4 Department of Pharmaceutical Sciences, College of Pharmacy and Pharmaceutical Sciences, Washington State University, Spokane, WA 99210, USA; 5 Department of Medicine, Pulmonary, Allergy, and Critical Care Division, School of Medicine, University of Pennsylvania, Philadelphia, PA 19104, USA; 6 Department of Systems Pharmacology & Translational Therapeutics, School of Medicine, University of Pennsylvania, Philadelphia, PA 19104, USA; 7 Department of Pharmacology, Center for Pharmacogenetics, School of Medicine, University of Pennsylvania, Philadelphia, PA 19104, USA

**Keywords:** oxidative stress, biomarkers, lung cancer, isoprostanes

## Abstract

Aim: 8-iso-prostaglandin F2α is a biomarker of lipid peroxidation, and one of the most commonly used measures of oxidative stress. It is an established biomarker of lung cancer risk. It is commonly measured by enzyme-linked immunosorbent assay. Given its importance, we developed a stable isotope dilution UPLC-tandem mass spectrometric method for the rapid determination of 8-isoprostane in blood. Methods: We tested the discriminatory capability of the method in 49 lung cancer patients, 55 benign lung nodule patients detected by chest X-ray, and 41 patients with chronic obstructive pulmonary disease (COPD) or asthma. Results: Significant differences were found in mean 8-isoprostane levels between the three groups (*p* = 0.027), and post-hoc tests found higher levels in the lung cancer patients than in patients with benign nodules (*p* = 0.032) and COPD/asthma (*p* = 0.014). The receiving operating characteristic area under the curve (AUC) was 0.69 for differentiating the lung cancer group from the benign nodule group, and 0.7 for differentiating from the COPD/asthma group. Conclusions: The UPLC-MS/MS-based method is an efficient analytical tool for measuring 8-isoprostane plasma concentrations. The results suggest exploring its utility as a marker for early lung cancer screening.

## 1. Introduction

Oxidative stress is caused by the imbalance between the generation of reactive oxygen species (ROS) and antioxidant responses [1]. Oxidative stress contributes to the pathogenesis of pulmonary and other diseases. The lung, in particular, is exposed to *exogenous* ROS from tobacco smoke and other pollutants, which can lead to pulmonary inflammation and the generation of *endogenous* ROS [2]. The cumulative effect of sustained oxidative stress includes chronic inflammatory responses such as asthma, COPD, and severe lung injury [3]. 8-iso-prostaglandin F2α is a product of lipid peroxidation that is often considered the best marker of oxidative stress [4,5,6]. Formed endogenously, it can be measured in various body fluids, including urine, plasma, exhaled breath condensate, amniotic fluid, saliva, and in tissues [7,8]. Elevated levels of 8-isoprostane were reported in patients with pulmonary diseases, including asthma [9,10,11,12], chronic obstructive pulmonary disease (COPD) [13,14], and cystic fibrosis [15,16]. Lung cancer is also thought to be triggered by inflammatory responses to environmental stimuli. Elevated urine 8-isoprostane levels at baseline were associated with a subsequent increased risk of lung cancer in the Shanghai Cohort Study [17] and in the German ESTHER cohort [18]. However, the precision of measurements of endogenous oxidative stress levels are often less than optimal due to issues in the detection methods [7]. The most common analytical methods for 8-isoprostane are enzyme-linked immunosorbent assay (ELISA), gas-chromatography mass spectrometry (GC-MS), and liquid chromatography mass spectrometry (LC-MS). Considering that there may be significant cross-reactivity with the multiple isomers of COX-derived prostaglandins, ELISA may not be the most precise method [19]. MS-based methods are more accurate and reliable, but more demanding [20]. Urine is the most widely used fluid for the measurement of 8-isoprostanes because it is the least invasive, but also sufficiently stable for clinical use [21]. A method for the urinary determination of 8-isoprostane was previously described [22]. Although the collection of blood is more invasive, it is routinely collected for research and diagnostic testing, especially for the collection and analysis of DNA. The aim of the current study, given the importance of 8-iso-prostaglandin F2α as a biomarker of lipid peroxidation and one of the most commonly used measures of oxidative stress, was to develop a stable isotope dilution UPLC-tandem mass spectrometric method for the rapid determination of 8-isoprostane in blood. We tested its discriminatory ability in blood from lung cancer cases and benign pulmonary conditions.

## 2. Materials and Methods

The data come from a larger multi-center case-control study conducted at Fox Chase Cancer Center, Penn State University, Temple University Hospital, and the University of Pennsylvania Medical Center to determine gene–environment interactions for lung cancer [23]. The current analysis was based on data and samples from the Penn State site. In brief, subjects were recruited from 2008 to 2011 at the Pennsylvania State Health Milton S. Hershey Medical Center (Hershey, PA) and PinnacleHealth (Harrisburg, PA); were between the ages of 40 and 70; and were either current or former smokers. All cases were newly diagnosed patients with lung cancer recruited from oncology clinics and practices. Eligible subjects were identified by weekly clinic scheduling logs by a study coordinator who further reviewed their medical and pathology reports to confirm the diagnosis, including histological confirmation. Control subjects were frequency-matched to cases by age (within five years) and sex. Controls were patients without lung cancer recruited from the Penn State and PinnacleHealth Departments of Medicine (Penn State, Division of Pulmonary, Allergy and Critical Care Medicine). Controls had nonmalignant conditions, including chronic obstructive pulmonary disease (COPD), asthma, or other pulmonary conditions, including benign lung nodules diagnosed by chest X-ray or conventional CT scan. In total, there were 335 cases and 398 controls. 

For the current sub-study, we selected all subjects with benign lung nodules (*n* = 58), and a sample of 49 lung cancer patients with non-small cell carcinoma frequency matched by age (within 5 years) and sex. We also selected 41 frequency matched controls with pulmonary conditions. The ages of the pulmonary controls were younger, and the matching criteria were extended to within 10 years. 

Information on subject characteristics was abstracted from the electronic medical records. Subjects also completed a standardized baseline questionnaire administered by the coordinator during their clinic visit, which contained items on medical history and socio-demographics, including race, ethnicity, years of education, marital status, medical history, and alcohol consumption. Questions on smoking history included ever having smoked 100 cigarettes during their lifetime, current and past smoking, and cigarettes per day. Former smokers were subjects who had smoked at least 100 cigarettes in their lifetime, but who had quit smoking at the time of interview. Occupational history was recorded using job codes. Subjects were asked if they had ever been exposed to known or suspected occupational lung carcinogens, including asbestos, coal dust, wood dust, diesel exhaust, and others. This study was performed according to the Code of Ethics of the World Medical Association, and was approved by the Institutional Review Board at Pennsylvania College of Medicine. All subjects completed an informed consent form. 

### 2.1. Sample Collection

Blood was collected from each participant, with plasma obtained through collection in purple-top tubes containing EDTA (8 mL). Samples were centrifuged, and 0.5 mL of plasma was transferred into 12 cryotubes and immediately labelled and stored at −80 °C.

### 2.2. Chemicals

8-isoprostane (8-iso prostaglandin F2α, 8-*epi* PGF_2α_) and 8-isoprostane-d^4^ were purchased from Cayman Chemicals (Ann Arbor, MI, USA). Acetic acid (AA) was purchased from Sigma-Aldrich (St. Louis, MO, USA) and formic acid was purchased from J. T. Baker (Phillipsburg, NJ, USA). Optima LC-MS grade water, acetonitrile, methanol, and other chemicals were purchased from Fisher Scientific (Fairlawn, NJ, USA). 

### 2.3. Sample Preparation

8-isoprostane standard stock solution (1 mg/mL) was prepared in ethanol. A range of working concentrations from 0.25 ng/mL to 10^4^ ng/mL was prepared from the 8-isoprostane stock solution by serial dilution with methanol. An available 8-isoprostane-d^4^ stock solution (100 ug/mL methyl acetate; Cayman Chemicals (Ann Arbor, MI, USA)) was diluted with methanol to 10 ng/mL to generate an internal standard working solution. Standard samples were prepared by spiking 3 uL of standard working solution and 3 uL internal standard working solution into 24 uL 50% methanol containing 0.1% formic acid. The calibration curve was performed from 25 pg/mL to 1000 ng/mL.

After spiking 3 µL internal standard working solution into 300–1000 µL plasma, samples were diluted with 400 µL of 5% methanol, followed by 400 µL of 1% formic acid to acidify. Samples were vortexed and centrifuged at 4 °C, 4000 rpm for 10 min before loading onto Strata-X (30 mg/1 mL) cartridges (Phenomenex, Torrance, CA, USA) for solid phase extraction (SPE). The SPE cartridges were preconditioned by 1 mL methanol and were then equilibrated with 1 mL 0.1% formic acid. After loading the samples, the SPE cartridges were washed with 0.1% formic acid and hexane. The cartridges were dried before the analytes were eluted with 1 mL of 100% ethyl acetate. The eluents were then dried by Speed Vac before reconstitution with 30 µL 50% methanol containing 0.1% formic acid. The reconstituted samples were centrifuged at 4 °C, 4000 rpm for 10 min before 10 µL aliquots were injected into the high-performance liquid chromatography with tandem mass spectrometry (UPLC-MS/MS) system. 

### 2.4. HPLC-MS/MS Analysis

The samples were analyzed using a Sciex QTRAP 6500+ MS coupled with a Sciex EXion HPLC separation system (Waltham, MA, USA). A 1.7-µm Acquity UPLC BEH C18 analytical column (1.0 × 100 mm; Waters, Dublin, Ireland) was used to separate 8-isoprostane from its metabolites and isomers, as well as other plasma components. The gradient elution was conducted using a flow rate of 0.15 mL/min with the following conditions: 7 min in 35% mobile phase B (0.1% acetic acid in acetonitrile: methanol (50:50)) and 65% solvent A (0.1% acetic acid in water), a linear gradient to 100% mobile phase B in 1 min, and followed by 100% mobile phase B for 1.5 min to flush the column before equilibration under the initial conditions. 

The Sciex QTrap 6500+ MS was equipped with an electrospray ionization probe operated in negative mode. The decluster potential (DP) was −86 V, the entrance potential (EP) was −10 V, the collision energy (CE) was −34 V, and the collision cell exit potential (CXP) was −15 V, whereas the curtain gas (CUR) was 35 L/h, and the collision gas (CAD) was medium. The ionSpray voltage was −4500 V, the temperature was 530 °C, gas 1 was 24 L/h, and gas 2 was 30 L/h.

The multiple reaction monitoring mode (MRM) was used to analyze and quantify 8-isoprostane and 8-isoprostane-d4, with the transitions of *m*/*z* 353.1 > 193.2 for 8-isoprostane and 357.1 > 197.2 for 8-isoprostane-d4. All peaks were integrated and quantified by Sciex OS 1.5 software. 

### 2.5. Statistical Analysis

Descriptive statistics of subject characteristics included means (±SD) and frequency counts. For 8-isoprostane levels, we conducted a one-way analysis of variance (ANOVA) followed by a Fisher’s least significant differences (LSD) post-hoc-test to assess the differences of 8-isoprostane levels between the lung cancer, benign nodule, and control groups, with a *p*-value < 0.05 considered statistically significant. The area under the receiver operating characteristic curve (AUC) was calculated for 8-isoprostane levels between the lung cancer and benign nodule groups, and displayed graphically. Multiple linear regression was used to examine the effect of gender (male vs. female, age at diagnosis, smoking history (current vs former), and occupational exposures (e.g., wood dust, asbestos, coal, diesel; ever exposed vs. never exposed) on plasma 8-isoprostane levels. Assumptions of linear regression were checked by regression diagnostics, including examining the scatter plot of studentized residuals against the predicted values. The Durbin–Watson test was used to analyze the residuals, with a value near 2.0 indicating no autocorrelation. IBM SPSS Statistics (version 23.0) was used for all the statistical analyses. The AUC for 8-isoprostane levels between lung cancer patients and controls was also calculated. 

## 3. Results

### 3.1. Study Sample Description

Table 1 shows the demographic characteristics of the subjects, including age, sex, and smoking status. In addition, among the cases, 11 reported past occupational exposure to diesel exhaust, followed by 10 who reported exposure to wood dust, 7 to coal, and 7 to asbestos. These were not mutually exclusive, as some subjects reported multiple exposures. Among the benign nodule group, nine reported exposure to diesel exhaust, nine to wood dust, seven to coal, and eleven to asbestos. 

### 3.2. Quantification of Plasma 8-Isoprostane

The analytical method developed was specific and selective for the measurement of plasma 8-isoprostane; no significant endogenous interfering components were found at the retention time of the analyte. The linear range of the method was from 25 pg/mL to 500 ng/mL for 8-isoprostane to cover the distribution of the concentrations from all plasma samples. The limit of quantification (signal/noise ≥ 10) was 2.5 pg/mL, and the limit of detection (signal/noise ≥ 3) was 0.8 pg/mL. The limit of quantification and limit of detection were (2.5 ± 0.2) pg/mL, N = 6, RSD = 8%; and (0.8 ± 0.1) pg/mL, N = 6, RSD = 12.5%.

### 3.3. Sample Preparation

A solid phase extraction procedure was used for the extraction of 8-isoprostane from human plasma, and, after comparing with other SPE cartridges, Phenomenex Strata-X (30 mg/1 mL) sorbent was used because of the high recovery. Plasma samples were acidified by adding 400 μL 1% formic acid before loading, and the cartridge was washed with 0.1% formic acid and hexane, which did not result in significant loss of the analyte, but helped to clean up the matrix. 8-isoprostane was eluted with ethyl acetate, which is easy to evaporate before reconstitution. 

### 3.4. UPLC and MS Analysis of 8-Isoprostane 

As 8-isoprostane is a weekly acidic compound, it can be deprotonated [M-H]^−^ at *m*/*z* 353.1 in the ESI source and detected in negative MRM mode. MS/MS was used to fragment the molecular ion to form a specific product ion at *m*/*z* 193.2, and, thus, MRM transition of *m*/*z* 353.1 → 193.2 was selected for quantification. 

**Figure 1:**
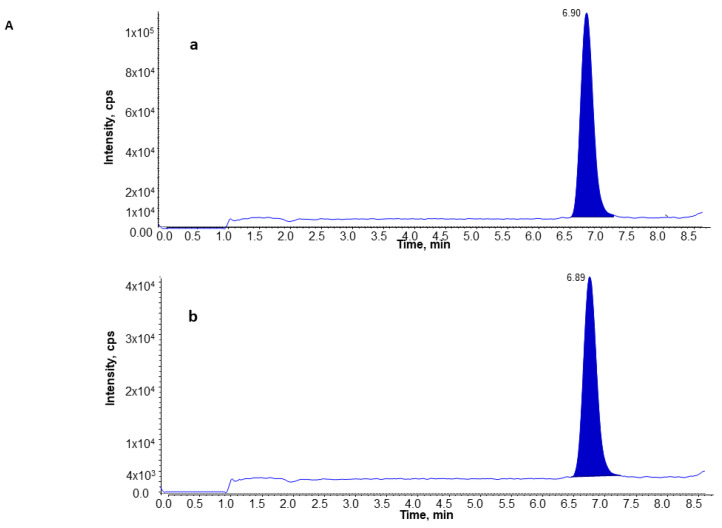

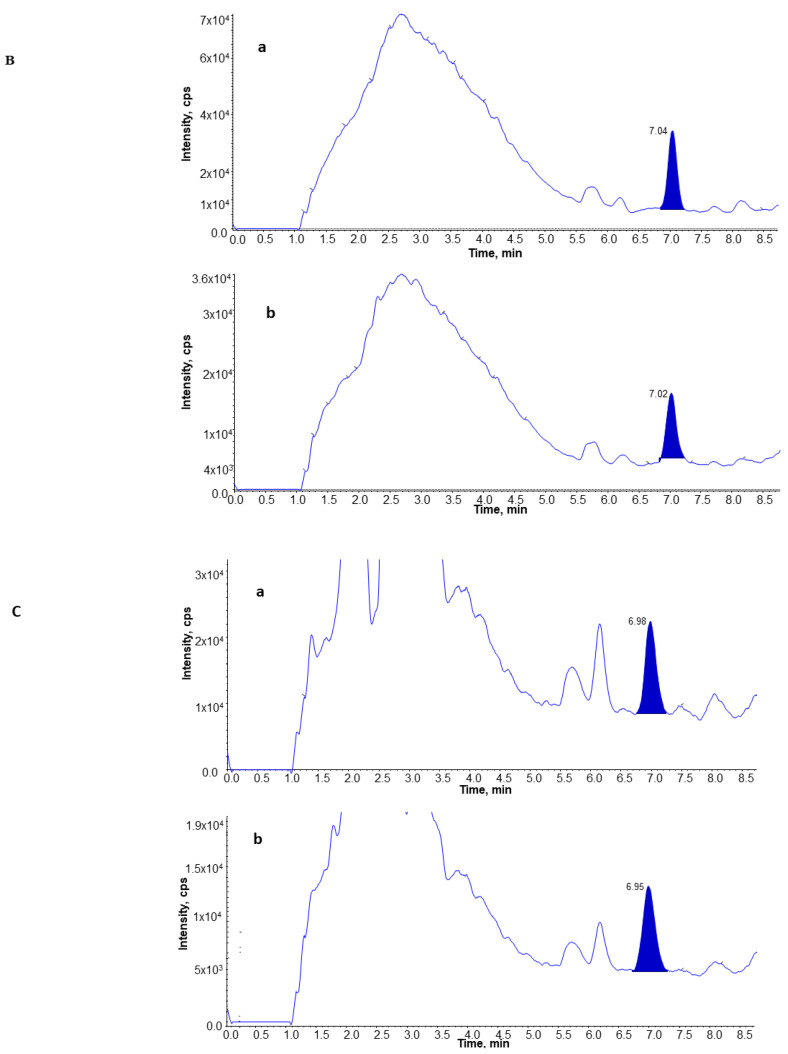

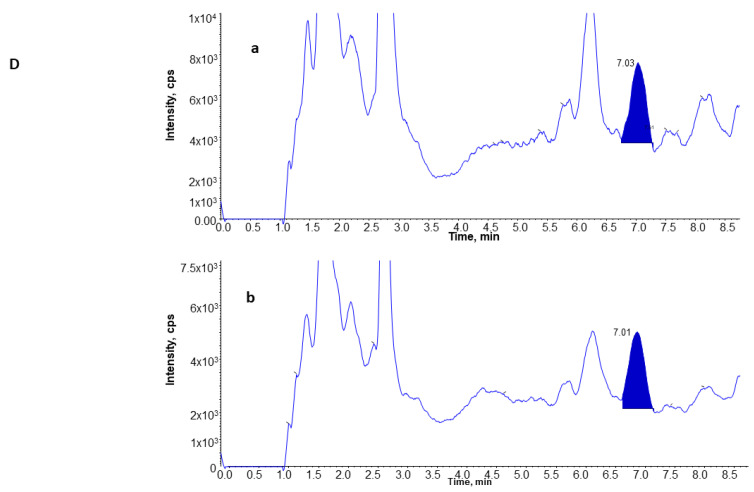
Chromatography of 8-isoprostane and its internal standard, 8-isoprostane-d4, in standard samples (**A**), in subjects with lung benign nodules (**B**), in lung can-cer patients (**C**), and in healthy controls (**D**). Panel a is for 8-isoprostane and panel b is for 8-isoprostane-d4 in each sample.

Due to the presence of other isoprostanes formed endogenously, a chromatography separation of 8-isoprostane from the other classes of isomers was necessary to avoid interference. As described in Materials and Methods, a seven-minute isocratic UPLC program using a 1.7-μm C18 column showed high resolution and efficiency to separate 8-isoprostane from other endogenous plasma compounds, and acetic acid was used as a buffer additive to enhance the sensitivity. As shown in Figure 1, the 8-isoprostane was eluted at 6.9 min in standard samples (Panel A), and a similar pattern was observed in plasma from the subjects with benign nodules (Panel B), lung cancer (Panel C), and in controls (Panel D), whereas for 8-isoprostane-d4, the internal standard in plasma was eluted out at the same time. 

### 3.5. Levels of 8-Isoprostane in Plasma from Subjects with Lung Cancer, Benign Lung Nodules, and from Controls

The mean (±SD) level of plasma 8-isoprostane for samples from all subjects was 9.41 ± 5.37 pg/mL (range 1.10 to 42.22 pg/mL). For the lung cancer group, the mean (±SD) level was 11.05 ± 4.29 pg/mL (range 3.73 to 29.16 pg/mL). For the benign nodule group, the mean (±SD) level was 8.84 ± 7.0 pg/mL (range 1.10 to 42.22 pg/mL). For the control group, the mean (±SD) level was 8.26 ± 2.98 pg/mL (range 2.88 to 15.74 pg/mL).

### 3.6. Comparison of 8-Isoprostane Levels between Subjects with Lung Cancer and Benign Lung Nodules, and Controls

**Figure 2:**
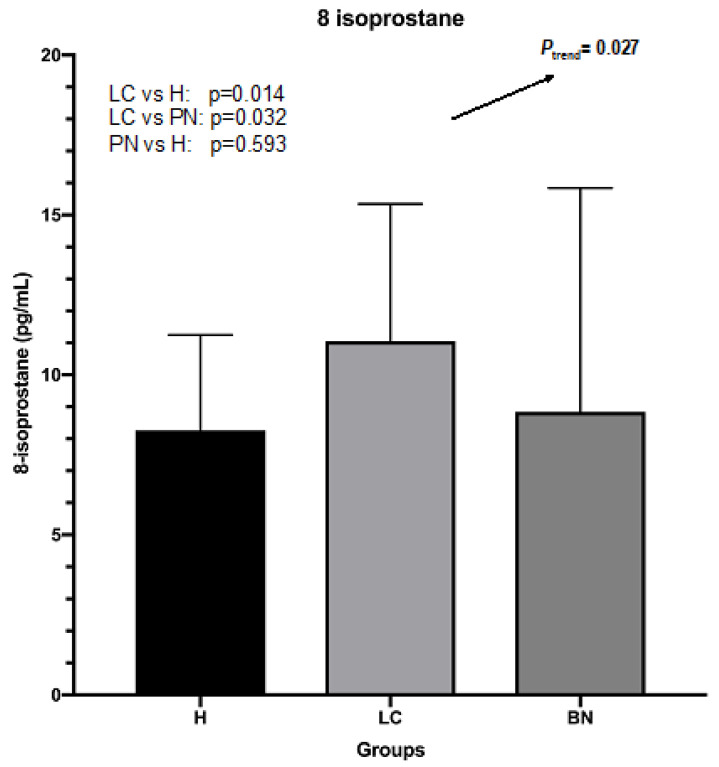
Levels of plasma 8-isoprostane in lung cancer, benign nodules, and control groups.

In ANOVA analysis, the *P*_trend_ for the three groups is 0.027, which indicates a linear trend in the mean level of 8-isoprostane from the lung cancer group to the benign nodule group to the control group. The post-hoc LSD test showed that the mean levels were higher in the lung cancer group compared to the benign nodule group (*p =* 0.014) and compared to the control group (*p =* 0.032) (Figure 2). However, there was no statistically significant difference in levels between the benign nodule group and the control group (*p* = 0.593). 

**Figure 3:**
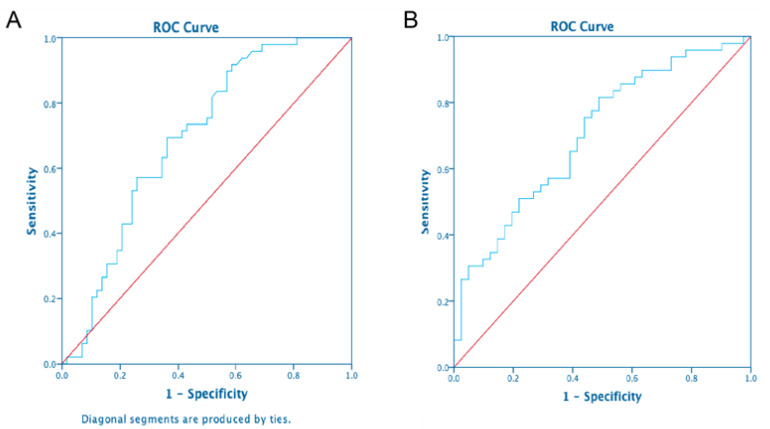
(**A**) Receiver operating characteristics (ROC) curve between lung cancer and benign nodules, (**B**) Receiver operating characteristics (ROC) curve between lung cancer and controls.

The AUC is 0.69, indicating that the level of plasma 8-isoprostane has diagnostic value for distinguishing the presence of lung cancer from benign nodules (Figure 3). In linear regression analysis, there was a significance difference in levels between the lung cancer and benign nodule group (*p* = 0.002) after adjustment for sex, age, and other potential confounders. The ROC that compared the lung cancer group to the controls had an AUC of 0.70. 

### 3.7. Predictors of 8-Isoprostane Concentrations

We further examined whether sex, age, smoking history, or occupational lung cancer risk factors could influence the levels of plasma 8-isoprostane. The adjusted R square was 0.17, and the *p*-value was 0.010. Disease status (*p*-value = 0.02) and current smoking (*p*-value = 0.03) were the only variables significantly correlated with the level of plasma 8-isoprostane. The scatter plot of studentized residuals and predicted values showed no pattern, indicating a good model fit. The variables were mutually independent (Durbin–Watson test value = 2.22).

## 4. Discussion

We developed a UPLC-MS/MS to evaluate the absolute and relative levels of 8-isoprostane in plasma, given that the measurement of inflammation and oxidative biomarkers in blood is standard practice for cancer research [24], where the simultaneous collection of the buffy coat layer in blood allows for genetic analysis. The method was applied to samples collected from subjects with malignant lung cancer, benign lung nodules, and in subjects without lung lesions who had pulmonary conditions. The results showed that the mean level of 8-isoprostane was significantly higher in the lung cancer group vs. both the benign nodule and the control group, with a significant trend of increasing plasma 8-isoprostane levels from pulmonary controls to subjects with lung benign nodules to subjects with lung cancer. The results indicate that oxidative stress, as measured by 8-isoprostane using the methods described, has a role in the pathogenesis of lung lesions. Studies using a variety of oxidative stress measures previously showed elevated concentrations in COPD compared to healthy individuals [25]. Our studies showed the highest levels of 8-isoprostane in lung cancer patients. This seems consistent with previous findings using other measures of oxidative stress, including malondialdehyde and oxidatively damaged DNA and proteins. These blood markers were also higher in lung cancer vs. COPD patients [26,27]. 

Tobacco smoke increases oxidative stress in humans from the large number of free radicals and other constituents, including carbonyls [28,29]. There was a significant association between smoking and plasma 8-isoprostane concentrations in the current study, which is consistent with other findings [13]. In addition, compared to former smokers, the levels of plasma 8-isoprostane were higher in current smokers. Since all the participants in the present study were either current smokers or former smokers, it was not possible to determine the relative levels of 8-isoprostane to nonsmokers. In the Shanghai cohort study [17], urine 8-isoprostane levels were lowest in never-smokers. 

Other factors reported to be associated with isoprostane levels included older age and female sex [30]. We did not observe an effect with these factors, although the age range was limited. We examined levels by exposure to occupational lung carcinogens, and did not observe any differences, although few were exposed to specific agents, and we also did not have information on exposure conditions and durations. 

Due to its relatively low concentrations, it is challenging to measure plasma 8-isoprostane. It was reported that, across the types of specimens, urine has the highest average concentration of free 8-iso-PGF_2α_ (1200 ± 600 pg/mL (1.3 ± 0.8 ng/mg creatinine) [31]). On average, ~100-fold less free 8-iso-PGF_2α_ was detected in plasma (45.1 ± 18.4 pg/mL) and exhaled breath condensate (EBC) (30.9 ± 17.2 pg/mL). 8-isoprostane in plasma has usually been measured by ELISA, and many previous studies have used ELISA to measure 8-isoprostane in urine or exhaled breath condensate [3,13,32,33]. 8-isoprostane belongs to a group of 64 isomers; thus, ELISA may not be the best way to definitively determine its concentration due to possible cross-reactivity between with related isomers [22]. More recently, MS methods have been described in mice plasma [34]. Methods from human samples have also been reported, although analytic details, including chromatograms, were not provided [35].

The strengths of the study include the UPLC-MS/MS method described here, which provides clear baseline separation of 8-isoprostane from all impurities and excellent sensitivity, with a quantification limit of 2.5 pg/mL (signal/noise ≥ 10) and a detection limit of 0.8 pg/mL (signal/noise = 3). As little as 300 μL plasma was sufficient, an important consideration for studies in which plasma volumes are limited. Another advantage is the wide linear range of detection, from as low as 25 pg/mL to 500 ng/mL, which encompassed the lower and upper plasma 8-isoprostane concentrations in this study. Compared to previous studies using ELISA, the MS-based method was more efficient and reproducible [36]. The use of a heavy-isotope-labeled internal standard for determining the analyte mitigated the potential problem of sample loss by calibrating the recovery rate [37]. Thus, the possible issue of cross-reactivity and the consequent unreliable results from using ELISA kits could be avoided. The use of gas chromatography (GC)/MS methods to determine levels of 8-isoprostane has previously been reported, and has indicated that they are precise and accurate compared to immunoassays [38,39]. However, the use of GC/MS is time-consuming and requires complicated SPE steps and derivatization reactions.

The limitations of the study include the fact that 8-isoprostane can be generated from ex vivo oxidation *of* arachidonic acid in the plasma during sample collection and storage, causing artificially high levels of measured plasma 8-isoprostane [40]. To minimize this effect in the present study, tubes containing EDTA were used for plasma processing and storage [41]. These samples were not previously thawed and refrozen, which would otherwise have contributed to sample oxidation. Still, the long-term storage of these samples likely resulted in a certain degree of oxidation, although it is expected that this would be similar for the three study groups.

Several studies have shown an association between relatively high concentrations of 8-isoprostane and lung disorders, including cancer, asthma, and COPD [5,17,25]. A multiethnic cohort study reported that, compared to the lowest tertile of urinary 8-isoprostane concentration, the second and third tertiles were associated with a higher lung cancer risk among men, but not among women [42].

Comparative studies of 8-isoprostane levels between malignant and benign lung nodules have not been reported. Benign lung nodules are often characterized by chronic inflammation due to infection from viruses or other pathogens. In our study, the levels of 8-isoprostane were similar in subjects with benign nodules and those with other inflammatory pulmonary conditions such as COPD. The highest oxidative stress levels were in the lung cancer patients. The current study was based on X-ray-detected pulmonary nodules, which are, on average, larger than those detected by LDCT. One potential utility of such a measurement is its ability predict the clinical outcomes and clinical management of patients with lung nodules. Blood-based protein markers are being studied as predictors of lung cancer in individuals undergoing low-dose computed tomography (LDCT) screening for lung cancer. Inflammation might also be a marker of malignant vs. benign lung nodules. Prospective studies of LDCT-detected nodules would be needed to determine if inflammation markers would have additional predictive power beyond existing clinical risk factors either at baseline or at subsequent screenings [43]. 

## 5. Conclusions

The aim of the current study was to develop a method for the measurement of 8-iso-prostaglandin F2α, a biomarker of lipid peroxidation and an established marker of lung cancer risk. It is commonly measured by enzyme-linked immunosorbent assay. Here, we developed a stable isotope dilution UPLC-tandem mass spectrometric method for the rapid determination in blood, and tested the discriminatory capability of the method in 49 lung cancer patients, 55 benign lung nodule patients detected by chest X-ray, and 41 patients with chronic obstructive pulmonary disease (COPD) or asthma. Significant differences were found in mean 8-isoprostane levels between the three groups. The UPLC-MS/MS-based method is an efficient analytical tool for measuring 8-isoprostane plasma concentrations. The results suggest exploring its utility as a marker for early lung cancer screening. The 8-isoprostane method developed here may be a useful biomarker for etiologic and outcome research.

## Figures and Tables

**Table 1:** Basic subject characteristics among cases and controls.

	Cases	Controls (Benign Nodules)	Controls (COPD)
Mean age	59.3 ± 7.4	59.9 ± 6.4	48.4 ± 6.38
Sex Males Females	20 (41%) 29 (59%)	24 (44%) 31 (56%)	22 (54%) 19 (465)
Smoking status Current Former	16 (33%) 33 (67%)		

## Data Availability

Data are available upon written request to the authors and the approval of data transfer agreements.
